# 15 weeks of soccer training increases left ventricular mass and improves indices of left ventricular diastolic function in previously sedentary, mildly hypertensive, middle-aged women

**DOI:** 10.1007/s00421-023-05399-7

**Published:** 2024-01-04

**Authors:** Tórur Sjúrðarson, Kasper Kyhl, Nikolai B. Nordsborg, Rudi Kollslíð, Lars Juel Andersen, Peter Krustrup, Magni Mohr

**Affiliations:** 1 Centre of Health Science, Faculty of Health Sciences, University of the Faroe Islands, Tórshavn, Faroe Islands; 2 Department of Nutrition, Exercise and Sports, University of Copenhagen, Copenhagen, Denmark; 3 Centre of Health Science, Department of Medicine, The Faroese National Hospital, Tórshavn, Faroe Islands; 4 Department of Cardiology, Copenhagen University Hospital Rigshospitalet, Copenhagen, Denmark; 5 Department of Cardiology, Zealand University Hospital, Roskilde, Denmark; 6 Department of Sports Science and Clinical Biomechanics, SDU Sport and Health Sciences Cluster (SHSC), University of Southern Denmark, 5250 Odense M, Denmark; 7 Danish Institute for Advanced Study (DIAS), University of Southern Denmark, Odense M, Denmark; 8 Sport and Health Sciences, College of Life and Environmental Sciences, University of Exeter, Exeter, UK

**Keywords:** Exercise training, Team sport, Cardiac function, Cardiac structure, Echocardiography

## Abstract

**Purpose:**

To investigate the impact of soccer training on cardiac adaptations in mildly hypertensive middle-aged women.

**Methods:**

Hypertensive premenopausal women (*n* = 41; age (mean ± SD): 44 ± 7 years; height: 166 ± 6 cm; weight: 78.6 ± 11.6 kg; body fat: 43.3 ± 5.2%) were randomized to soccer training (SOC, *n* = 21) or control (CON, *n* = 20). SOC performed three weekly training sessions for 15 weeks, whereas CON had no training or lifestyle changes during the same period. Cardiac structure and function were assessed by echocardiography pre-intervention and post-intervention.

**Results:**

Soccer training increased (*P* = 0.001) left ventricular mass index by 10% [95% CI 4; 15], while no changes occurred in CON (time × group interaction, *P* = 0.005). In addition, only SOC demonstrated a within-group increase (*P* = 0.01) of 8% [95% CI 2; 14] in left ventricular septum diameter. For markers of right ventricular remodelling, a within-group increase (*P* = 0.02) occurred for tricuspid annulus plane systolic excursion of 8% [95% CI 1; 14] in SOC only. Left atrial diameter index increased (*P* < 0.001) by 6% [95% CI 3; 10] after SOC, while it was unaffected in CON (time × group interaction, *P* = 0.02). For makers of diastolic function, SOC demonstrated a within-group increase (*P* = 0.02) in the average early diastolic mitral annulus velocity of 10% [95% CI 2; 19]. In addition, a reduction (*P* < 0.001) in mitral valve A velocity of − 19% [95% CI − 29; − 10] was observed following soccer training, which manifested in increased (*P* < 0.001) mitral valve *E*/*A* ratio of 34% [95% CI 16; 53] in SOC. No within-group changes were apparent in CON.

**Conclusion:**

In sedentary, mildly hypertensive, middle-aged women, 15 weeks of soccer training increases left ventricular mass and left atrial diameter and improves indices of left ventricular diastolic function.

## Introduction

In developed countries, cardiovascular disease (CVD) remains the leading cause of mortality (Townsend et al. 2022). The burden of CVD can be largely explained by means of the traditional risk factors affecting both genders, including smoking, elevated cholesterol levels, physical inactivity, overweight, diabetes and arterial hypertension (Jagannathan et al. 2019; Appelman et al. 2014). However, compelling evidence indicates robust and independent CVD risk factors that are exclusive to women (Sattar and Greer 2002; Rosano et al. 2007), including disorders of pregnancy, polycystic ovarian syndrome, premature menopause and menopause (Appelman et al. 2014; Harvey et al. 2015; Cho et al. 2020), which emphasizes the importance of gender-specific guidelines for CVD prevention and treatment.

In 1999, women-specific guidelines for CVD prevention were developed by the American Heart Association (AHA) (Mosca et al. 1999), which emphasized the importance of regular physical exercise as an adjunct to the pharmacological treatment and prevention of CVD. Specifically, the most recent women-specific guidelines from AHA (Arnett et al. 2019) recommend at least 150 min per week of moderate-intensity physical activity or 75 min of vigorous-intensity physical activity weekly. However, the optimal characteristics of the training programme to reduce CVD risk factors including health-beneficial changes in cardiac structure and function have not been clearly defined or identified.

Recent studies from our group indicate that soccer training can be an effective tool in the primary and secondary prevention of CVD by reducing numerous cardiovascular risk factors in both healthy and hypertensive individuals (Krustrup et al. 2018, 2010; Milanović et al. 2019; Andersen et al. 2010). For instance, Andersen et al. (2010) demonstrated that 16 weeks of soccer training induced substantial changes in cardiac dimensions and improved both left ventricular systolic and diastolic function in sedentary healthy premenopausal females, and Krustrup et al. (2010) reported that 16 weeks of recreational soccer training resulted in improved pulse pressure wave augmentation index, a marker of arterial stiffness in untrained healthy premenopausal women.

Thus, the main purpose of the present study was to investigate the effects of 15 weeks of recreational soccer training on cardiac structure and function in sedentary, mildly hypertensive, middle-aged women using conventional echocardiography, TDI and speckle tracking analysis. We hypothesized that the applied soccer training protocol would induce robust cardiac adaptations due to substantial cardiovascular loading during the training sessions.

## Materials and methods

### Compliance with ethical standards

The experimental protocol conformed to the standards set by the Declaration of Helsinki and was approved by the ethical committee of the Faroe Islands and the Sport and Health Sciences Research Ethics Committee at the University of Exeter, Exeter, United Kingdom. After being informed verbally and in writing of the experimental procedures and associated risks, all the participants gave their written consent to take part in the study.

### Participants

Sedentary, mildly hypertensive, premenopausal women (*n* = 41) with average (± SD) age, height, weight, body fat and systolic/diastolic blood pressure of 44 ± 7 years, 166 ± 6 cm, 78.6 ± 11.6 kg, 43.3 ± 5.2% and 137 ± 7/84 ± 5 mmHg, respectively, were recruited for the study.

### Experimental design

The study was designed as a randomized controlled trial and parts of the obtained data have been reported elsewhere (Mohr et al. 2014, 2015; Nordsborg et al. 2015). After an initial screening of 262 women volunteers, the participants were enrolled in the study based on selection criteria of a sedentary lifestyle for at least 2 years, mild hypertension (mean arterial pressure of 96–110 mmHg) and body mass index of at least 25 kg/m^2^. Moreover, solely premenopausal women were included. The participants were randomized into one of two intervention groups: a soccer training group (SOC, *n* = 21; age: 45 ± 5 years; height: 165 ± 7 cm; weight: 79.8 ± 12.8 kg) or a control group (CON, *n* = 20; age: 43 ± 4 years; height: 166 ± 6 cm; weight: 77.3 ± 10.4 kg). Soccer training was performed three times a week for 15 weeks, whereas CON had no training or lifestyle changes during the same period. Within 10 days before initiation of the intervention and 48–72 h after the last training session, all participants underwent echocardiography. Dietary intake was not controlled during the intervention, and the experimental days were not timed in relation to the menstrual cycle.

### Training intervention

SOC completed a total of 45 ± 3 training sessions over the 15-week intervention period. All SOC training sessions lasted 1 h and consisted of small-sided soccer games (4 vs 4 to 8 vs 8) as previously described (Krustrup et al. 2018). A trained soccer coach was present during all sessions to control the duration of the training and ensure competitive games. Heart rate was measured during a training session in the 1st and 15th week of training in order to describe the cardiovascular loading during the training sessions.

### Echocardiography

**Fig. 1:**
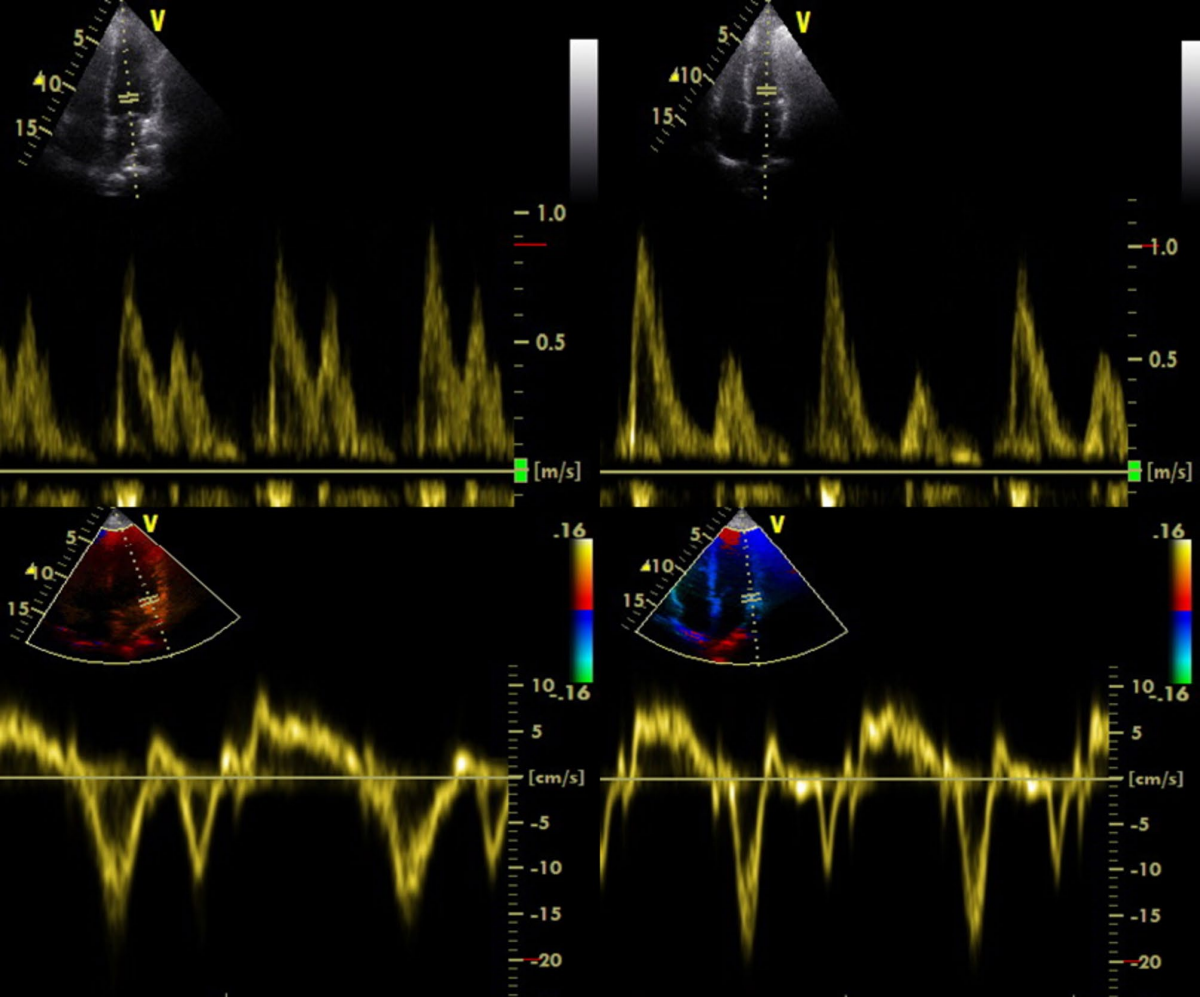
Representative Doppler echocardiographic images from before and after the intervention. Top: pulsed Doppler mitral inflow before (left) and after (right) the intervention indicating early (*E* wave) and late (*A* wave) diastolic transmitral flow. Bottom: pulsed-wave tissue Doppler measurement in the lateral mitral annulus before (left) and after (right) the intervention used to evaluate *E′*

Echocardiography was performed by a single experienced cardiologist, who was blinded to the experimental groups and pre-/post measurements on a GE Vivid E9 ultrasound (GE Medical System, Horten, Norway) with a 2.5 MHz transducer and subjects in the left lateral supine position as described previously (Hansen et al. 2013; Sjúrðarson et al. 2022). Analysis was performed offline using a commercially available software program (EchoPac, GE Medical System, Norway). In brief, two-dimensional (2D) ultrasound imaging, M-mode imaging, and pulsed-wave conventional and tissue Doppler measurements were produced, with measurements of LV and RV dimensions and interventricular septum thickness in parasternal long-axis 2D recordings at mid-ventricular level, and LV volumes and ejection fraction were calculated using Simpson’s biplane method (Schiller et al. 1989). Left atrium (LA) diameter was also measured in parasternal long-axis 2D recordings. Pulsed Doppler measurements of mitral inflow were performed in the apical four-chamber view to determine peak transmitral flows in early diastole (E) and during LA contraction (A), E/A ratio and deceleration time of early filling velocity curves. RV systolic function was assessed by TAPSE using M-mode echocardiography in the apical four-chamber view and TAPSE measured as the total displacement of the tricuspid annulus in the longitudinal direction from end diastole to end systole. In addition, pulsed-wave tissue Doppler imaging was performed in four-chamber apical projections of the medial and lateral myocardial wall to assess average early diastolic mitral annulus velocity (E′) (Sjúrðarson et al. 2022). For speckle tracking analysis, 2D images recorded with a frame rate of > 80 Hz from the standard apical view were analysed to track movements of “speckles” over the cardiac cycle. LV global longitudinal strain was calculated as an average of maximal systolic strains in all myocardial segments determined from apical projections as performed previously (Leitman et al. 2004; Reisner et al. 2004). Examples of representative original echocardiography images are shown in Fig. 1. Finally, indexed values were adjusted for body surface area (BSA) calculated with the Mosteller equation (BSA = square root of the height (cm) multiplied by the weight (kg) divided by 3600).

### Statistical analysis

Data are presented as means with 95% confidence intervals. Specific statistical analyses (using SPSS v. 27.0.0, IBM) were applied to answer the primary hypothesis. Continuous endpoints were analysed by a linear mixed model repeated measures approach by means of the SPSS mixed method (Cnaan et al. 2005). ‘Participant number’ specified random variation, and a repeated effect for time was included in the model. Main effects for ‘time’ (pre vs. post), ‘group’ (SOC and CON) and ‘time’ × ‘group’ interactions were further explored by Sidak-adjusted pairwise comparisons. Homogeneity of residual variance and the normality of the residuals were visually checked for all of the obtained data. The level of statistical significance was set at *P* < 0.05.

## Results

### Cardiac adaptions

All obtained cardiac measures were similar between SOC and CON at baseline (Table 1).

**Table 1:** Cardiac adaptations to 15 weeks of soccer training (SOC, *n* = 21) compared to a habitually active control group (CON, *n* = 20)

Outcome	Intervention						Main effects		
	SOC			CON					
	Pre	Post	*n*	Pre	Post	*n*	Time × group	Time	Group
Left ventricular adaptations									
LVDmass (g)	133 [120;146]	145 [132;157]**	19	137 [124;149]	138 [125;151]	19	*P* < 0.05	*P* = 0.01	*P* = 0.87
IVSd (cm)	0.78 [0.73;0.84]	0.84 [0.79;0.89]*	19	0.81 [0.75;0.86]	0.82 [0.77;0.87]	19	*P* = 0.20	*P* = 0.03	*P* = 1.00
IVSdI (cm/m^2^)	0.41 [0.38;0.44]	0.45 [0.42;0.48]*	19	0.43 [0.40;0.46]	0.44 [0.41;0.47]	19	*P* = 0.16	*P* = 0.03	*P* = 0.83
LVIDd (cm)	4.83 [4.67;4.98]	4.85 [4.69;5.00]	19	4.76 [4.60;4.91]	4.77 [4.62;4.93]	19	*P* = 0.93	*P* = 0.55	*P* = 0.50
LVIDdI (cm/m^2^)	2.53 [2.47;2.60]	2.57 [2.50;2.64]	19	2.53 [2.46;2.60]	2.53 [2.46;2.60]	19	*P* = 0.41	*P* = 0.28	*P* = 0.66
LVIDs (cm)	3.12 [2.97;3.27]	3.12 [2.97;3.27]^#^	18	2.92 [2.76;3.07]	2.88 [2.73;3.04]	17	*P* = 0.72	*P* = 0.62	*P* = 0.03
LVIDsI (cm/m^2^)	1.65 [1.56;1.73]	1.66 [1.58;1.74]	18	1.56 [1.47;1.64]	1.54 [1.45;1.62]	17	*P* = 0.52	*P* = 0.84	*P* = 0.06
Right ventricular adaptations									
RVIDd (cm)	2.77 [2.60;2.94]	2.72 [2.55;2.89]	18	2.82 [2.65;2.99]	2.81 [2.64;2.98]	18	*P* = 0.70	*P* = 0.55	*P* = 0.52
RVIDdI (cm/m^2^)	1.45 [1.36;1.54]	1.44 [1.35;1.53]	18	1.50 [1.42;1.59]	1.50 [1.41;1.58]	18	*P* = 1.00	*P* = 0.74	*P* = 0.31
TAPSE (cm)	2.30 [2.16;2.44]	2.48 [2.34;2.62]*	18	2.34 [2.20;2.48]	2.39 [2.25:2.53]	19	*P* = 0.22	*P* = 0.03	*P* = 0.81
Diastolic function									
MV *E* (m/s)	0.71 [0.66;0.77]	0.76 [0.70;0.81]	19	0.77 [0.71;0.82]	0.78 [0.72;0.83]	19	*P* = 0.38	*P* = 0.18	*P* = 0.32
MV *A* (m/s)	0.57 [0.52;0.63]	0.46 [0.41;0.52]***,^#^	19	0.60 [0.55;0.66]	0.56 [0.51;0.62]	19	*P* = 0.08	*P* < 0.001	*P* = 0.08
MV DT (ms)	170 [153;186]	176 [160;193]	19	183 [166;199]	195 [178;211]	19	*P* = 0.75	*P* = 0.25	*P* = 0.07
* E*/*E′* Septal	8.89 [7.82;9.97]	8.09 [7.01;9.16]	18	8.78 [7.73;9.82]	9.01 [7.96;10.06]	19	*P* = 0.18	*P* = 0.54	*P* = 0.45
*E*/*E′* Lateral	6.72 [6.01;7.44]	6.40 [5.69;7.11]	18	6.04 [5.34;6.73]	5.72 [5.02;6.41]	19	*P* = 0.99	*P* = 0.19	*P* = 0.12
LAd (cm)	3.35 [3.13;3.57]	3.55 [3.33:3.77]**	15	3.62 [3.41;3.83]	3.64 [3.43;3.85]	16	*P* = 0.03	*P* = 0.01	*P* = 0.23
Systolic function									
GLS (%)	− 17.6 [− 18.7;− 16.5]	− 18.2 [− 19.3;− 17.2]	13	− 18.1 [− 19.2;− 17.0]	− 19.1 [− 20.1;− 18.0]	13	*P* = 0.65	*P* = 0.06	*P* = 0.31
LVEF (%)	64.0 [61.2;66.8]	64.6 [61.8;67.5]^#^	18	67.9 [65.0;70.1]	69.4 [66.5;72.3]	17	*P* = 0.70	*P* = 0.35	*P* = 0.01

If a significant main effect of time, group or time × group existed, the result of the post hoc analysis is indicated by *P < 0.05, **P < 0.01, ***P < 0.001 for within-group change from baseline, ^#^*P* < 0.05 compared with CON. Values are presented as means with 95% confidence intervals from a repeated measures linear mixed model with time, group and time × group as explanatory variables.

*LVDmass:* left ventricular diastolic mass, *IVDs:* left ventricular septum diameter, *IVDsI:* left ventricular septum diameter adjusted for body surface area (BSA), *LVIDd:* left ventricular internal dimension at end diastole, *LVIDdI:* left ventricular internal dimension at end diastole adjusted for BSA, *LVIDs:* left ventricular internal dimension at end systole, *LVIDsI:* left ventricular internal dimension at end systole adjusted for BSA, *RVIDd:* right ventricular internal dimension at end diastole, *RVIDdI:* right ventricular internal dimension at end diastole adjusted for BSA, *TAPSE:* tricuspid annulus plane systolic excursion, *MV E Vel:* mitral valve *E* velocity, *MV DT:* mitral valve deceleration time, *MV A Vel:* mitral valve *A* velocity, *E/E′ septal*: ratio of septal *E* to *E′*, *E/E′ lateral*: ratio of lateral *E* to *E′*, *Lad:* left atrial diameter, *GLS:* global longitudinal strain, *LVEF:* left ventricular ejection fraction

### Left ventricular remodelling

**Fig. 2:**
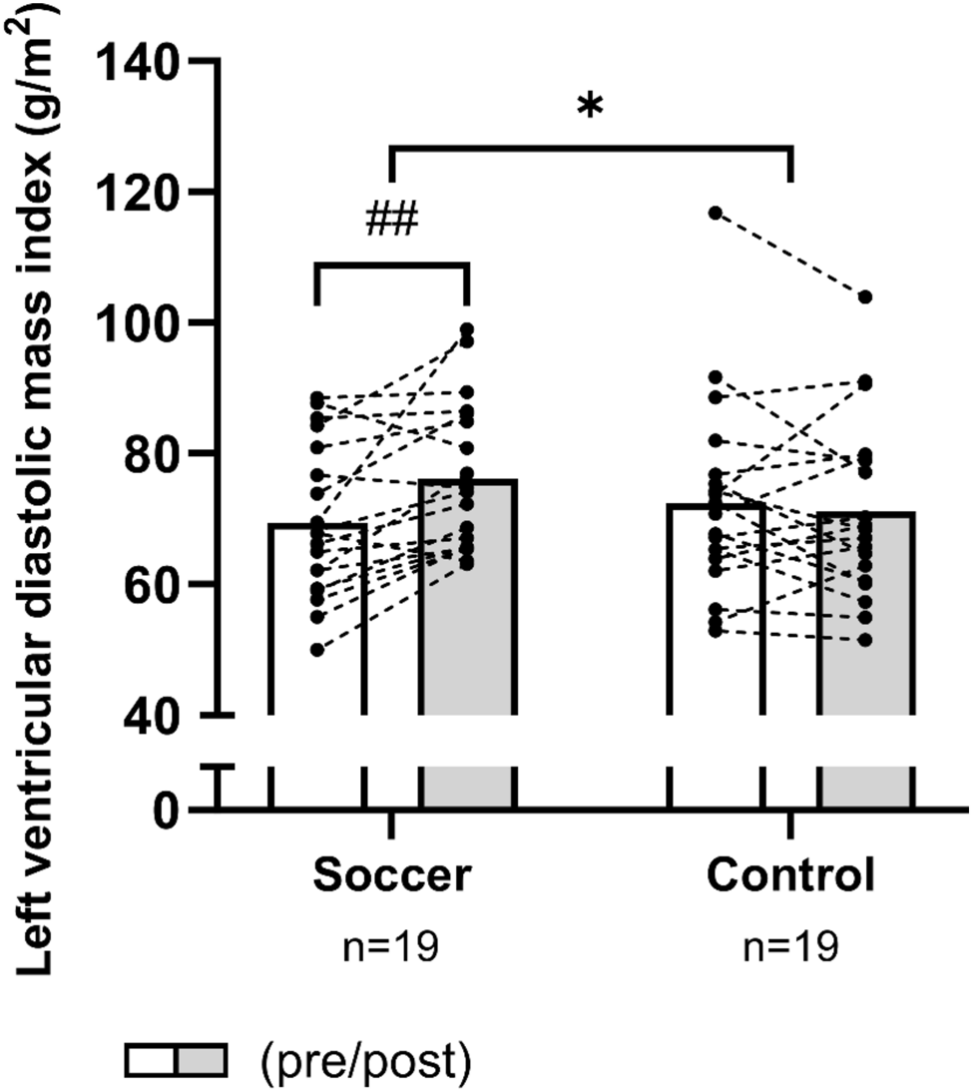
Mean values for left ventricular diastolic mass index in histograms with individual participants as dotted lines in the soccer group and the control group measured pre-intervention (white bars) and post-intervention (grey bars). *denotes a “time × group” interaction at *P* < 0.05. The result of the post hoc analysis is indicated by ^##^*P* < 0.01 compared with pre-intervention

Soccer training increased (*P* = 0.001) left ventricular mass index by 10% [4;15], whereas no changes were observed in CON (time × group, *P* = 0.005; Fig. 2). In addition, a main effect of time was observed for left ventricular septum diameter index. The pairwise comparison revealed a within-group increase (*P* = 0.01) of 8% [2;14] in left ventricular septum diameter index after SOC only (Table 1), but no significant between-group effect existed (time × group, *P* = 0.16; Table 1). No within-group or between-group change was observed for left ventricular internal dimension index at end diastole or end systole (Table 1).

### Right ventricular remodelling

No time × group interaction was observed for markers of right ventricular adaptations, but a main effect of time existed for tricuspid annulus plane systolic excursion. The post hoc analysis revealed a within-group increase (*P* = 0.02) in tricuspid annulus plane systolic excursion of 8% [1; 14] in SOC only (Table 1).

### Markers of diastolic function

**Fig. 3:**
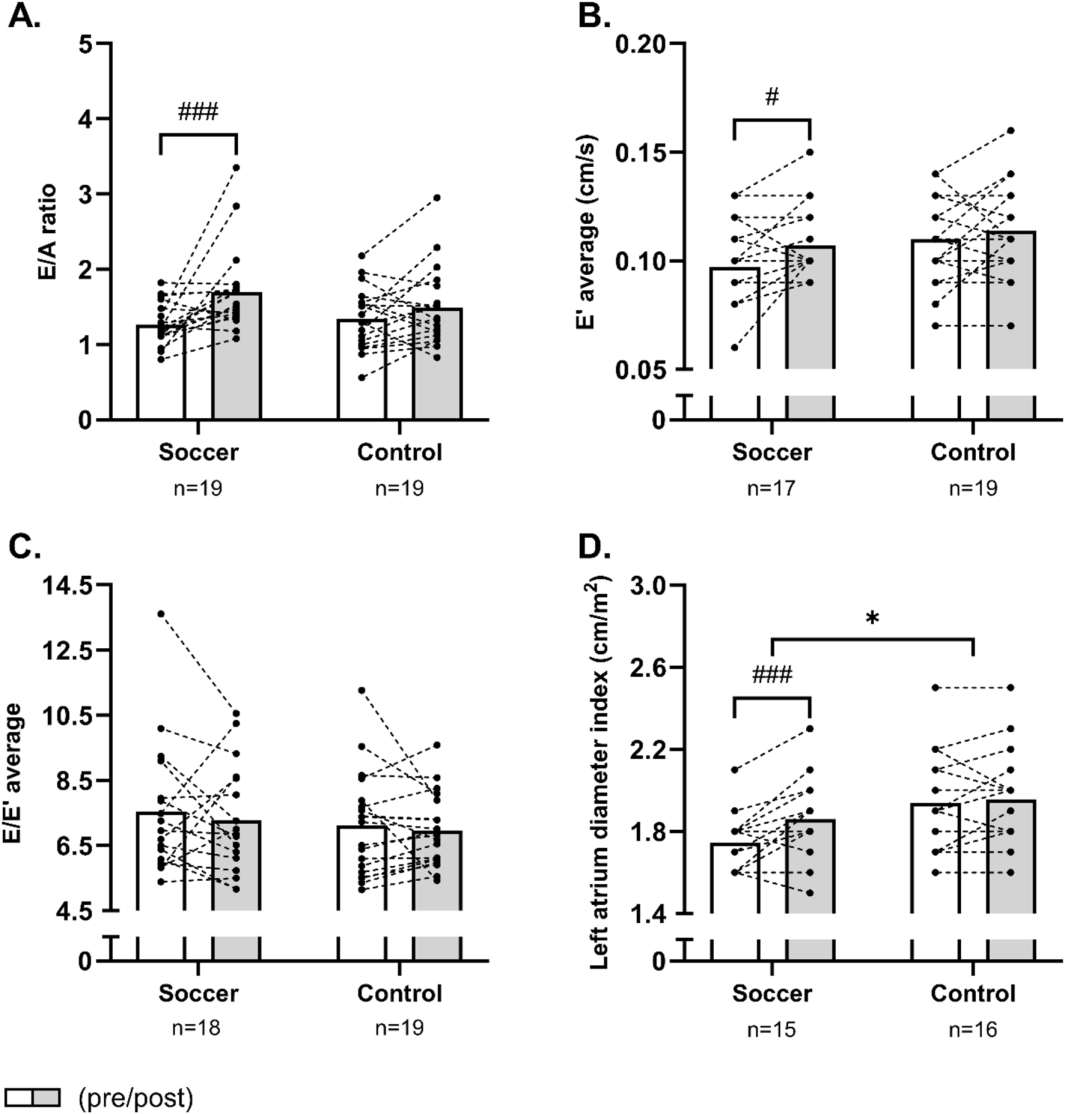
Mean values for the mitral valve *E*/*A* ratio (**A**), the average early diastolic mitral annulus velocity (**B**), the ratio of average *E* to *E′* (**C**) and the left atrial diameter index (**D**) in histograms with individual participants as dotted lines in the soccer group and the control group measured pre-intervention (white bars) and post-intervention (grey bars). *denotes a “time × group” interaction at *P* < 0.05. The result of the post hoc analysis is indicated by ^#^*P* < 0.05, ^###^*P* < 0.001 compared with pre-intervention

For markers of diastolic function, a main effect of time existed for the mitral valve *E*/*A* ratio, the average early diastolic mitral annulus velocity *E′* and the mitral valve *A* velocity. Specifically, the pairwise comparisons revealed a within-group increase (*P* < 0.001) in the mitral valve *E*/*A* ratio of 34% [16;53] (*P* < 0.001) and in the average early diastolic mitral annulus velocity *E′* of 10% [2;19] (*P* = 0.02) in SOC only, but no statistical differences between groups existed (Fig. 3A, B). Furthermore, a reduction (*P* < 0.001) in mitral valve A velocity of − 19% [− 29; − 10] was observed after SOC only, resulting in a 19% [4;34] lower (*P* = 0.02) mitral valve A velocity in SOC compared to CON post-intervention (Table 1). Finally, soccer training increased (*P* < 0.001) left atrial diameter index by 6% [3;10], whereas it was unaffected in CON (time × group, *P* = 0.02; Fig. 3D).

### Markers of systolic function

No between-group or within-group effect existed for global longitudinal strain or left ventricular ejection fraction (Table 1).

## Discussion

The present study investigated the effect of 15 weeks of soccer training on cardiac function and structure in middle-aged, sedentary, hypertensive women. Notably, 15 weeks of soccer training induced substantial increases in left ventricular mass and left atrial diameter compared to habitually active controls. In addition, significant within-group adaptations were observed for left ventricular septum diameter, tricuspid annulus plane systolic excursion, the average early diastolic mitral annulus *E′*, mitral valve *A* velocity and mitral valve *E*/*A* ratio in response to the soccer training only. Thus, our findings, which are supported by long-term trials in men (Andersen et al. 2014), indicate beneficial effects of 15 weeks of soccer training on key components for cardiac health in middle-aged sedentary hypertensive women.

Several randomized controlled trials have shown broad-spectrum health effects of 3–4 months of soccer training for untrained women (Krustrup et al. 2018). These findings are additionally supported by meta-analysis evidence that demonstrated beneficial effects on cardiovascular disease risk factors (Milanović et al. 2019, 2015) and superior effects of complex or hybrid training regimes compared to conventional endurance, high-intensity interval and resistance training protocols (Batrakoulis et al., 2022). However, few studies have investigated the impact of recreational soccer training on cardiac health.

The applied soccer training intervention effectively increased left ventricular mass compared to the control group. This observation, in conjunction with the within-group increase in left ventricular septum diameter in SOC, indicates that 15 weeks of soccer training with 3 weekly 1-h sessions is potent enough to induce concentric cardiac remodelling in sedentary, mildly hypertensive women. In accordance with this, Arbab-Zadeh et al. (2014) demonstrated that 3 months of endurance training generates concentric remodelling, including a ~ 12% and ~ 15% increase in left ventricular mass and left ventricular mean wall thickness, respectively, in previously sedentary, healthy individuals. In addition, a ~ 6% increase in left ventricular posterior wall thickness has been reported after 16 weeks of soccer training in healthy, previously sedentary women (Andersen et al. 2010). In contrast, no training-induced change existed for the left ventricular internal dimension at end diastole in the present study, which may require long-term exercise protocols to expose. Indeed, Arbab-Zadeh et al. (2014) reported no significant change in left ventricular end-diastolic volume after 3 months of endurance training, but reported a significant 8% increase after 6 months of endurance training. In contrast, Andersen et al. (2010) reported a 13% and 12% increase in left ventricular end-diastolic volume and right ventricle diastolic diameter, respectively, after 16 weeks of soccer training in healthy, previously sedentary women.

Although no time × group interaction existed for tricuspid annulus plane systolic excursion, a marker of right ventricular systolic function (Meluzín et al. 2001; Aloia et al. 2016), it should be pointed out that tricuspid annulus plane systolic excursion increased after soccer training. Accordingly, Andersen et al. (2010) demonstrated a substantial 16% increase in tricuspid annulus plane systolic excursion in response to 16 weeks of soccer training in healthy, previously sedentary women, and a study in untrained men reported augmented tricuspid annular plane systolic excursion after 3 months of soccer training (Andersen et al. 2014). Studies have demonstrated that the time course of the adaptive response in different cardiac variables to exercise training differs (Arbab-Zadeh et al. 2014; Fujimoto et al. 2010). Thus, our training intervention may have been too short to induce a significant between-group interaction.

In relation to markers of diastolic cardiac function, the applied soccer training protocol provoked a pronounced increase in left atrial diameter compared to the control group. Enlarged atrial diameter is an expected adaptation to endurance exercise training in healthy individuals (Sjúrðarson et al. 2022; Mahjoub et al. 2019), but even though enlarged cardiac chamber volumes are seen as typical observations amongst endurance-trained athletes (Maron and Pelliccia 2006), the clinical impact of left atrial expansion in borderline hypertensive women is unknown. While no time × group effect was observed for any of the other markers of diastolic function, it should be noted that the soccer training induced marked within-group decreases in mitral valve *A* velocity and thereby an increase in mitral valve *E*/*A* ratio promoting a shift towards a more passive filling of the left ventricle during diastole in the soccer group. Furthermore, a within-group increase in *E′* supports an improved diastolic left ventricular function in the soccer group. In support, Andersen et al. (2010) reported that 16 weeks of soccer training in healthy, sedentary women induced substantial improvements in left ventricular diastolic function in terms of mitral valve *A* velocity, mitral valve *E*/*A* ratio and *E′*. Collectively, these findings indicate that short-term soccer training, and hence, the combination of fast accelerations in heart loading and time spent in high heart rate zones can induce health-beneficial adaptations in diastolic function. The observed adaptations were, however, relatively small, and future studies should aim to further elucidate the long-term clinical effects.

Although CVD accounts for the majority of deaths amongst women, epidemiological studies have shown that premenopausal women are somewhat protected against developing CVD compared to males, which is demonstrated by a delayed presentation of CVD (Rosano et al. 2007; Vitale et al. 2009; Khoudary et al. 2020; Kannel et al. 1976; Iorga et al. 2017). The later onset of CVD in premenopausal women is attributed at least in part to the levels of the female hormone, oestrogen, which confers cardioprotection through a plethora of mechanisms that reduce oxidative stress, augment angiogenesis and improve vascular function (Rosano et al. 2007; Iorga et al. 2017). In support, the incidence of CVD increases considerably after the menopause, which coincides with an increase in numerous traditional CVD risk factors, including elevated blood pressure, endothelial dysfunction and vascular inflammation (Rosano et al. 2007). Thus, studies investigating the impact on cardiac health in women approaching the menopause such as the present study are highly warranted. Despite a somewhat unclear adaptive response in some of the obtained cardiac measures, the present findings indicate that sedentary premenopausal women with moderate arterial hypertension can benefit from 15 weeks of soccer training with respect to both cardiac structure and function. Future studies using longer training interventions should explore this further. It should be noted that blood pressure, total body weight, body composition and blood lipid profile were also measured pre- and post-intervention, and these data have been reported elsewhere (Mohr et al. 2014). Briefly, 15 weeks of soccer training induced marked reductions in systolic blood pressure (− 12 mmHg) and diastolic blood pressure (-6 mmHg), whereas no changes were apparent in CON. The change in total body weight was not statistically different between groups, but the change in total body fat mass was significantly different in SOC (− 2.3 kg) compared to CON (0.4 kg), and the changes in both total cholesterol (SOC: − 0.4 mmol/L vs. CON: 0.1 mmol/L) and triglycerides (SOC: − 0.2 mmol/L vs. CON: 0.3 mmol/L) were statistically different between SOC and CON (Mohr et al. 2014).

Our study has some limitations. Firstly, the cardiovascular effects of exercise training in our population of sedentary, mildly hypertensive middle-aged women might affect other parameters than the ones examined, including vascular stiffness that has been proven to increase before the manifestation of left ventricular hypertrophic remodelling (Kyhl et al. 2021).

The strength of strain analysis is that it is an objective measure that is able to detect miniscule changes in cardiac performance on a continuous scale. However, only GLS was available in the present study. Radial, circumferential, and transverse strain, which might have added information to the present study, were not performed. Finally, adjustment for multiple tests using the Sidak correction might lead to an increased risk of type II errors, and thus, it should be taken into account that an actual effect might have been missed.

In conclusion, 15 weeks of soccer training induces marked increases in left ventricular mass and left atrial volume in middle-aged, sedentary, hypertensive women and promotes signs of improved left ventricular filling. Thus, the present findings indicate a substantial cardiac plasticity to short-term soccer training in sedentary, mildly hypertensive women.

## Data Availability

The data sets generated during and/or analysed during the current study are available from the corresponding author on reasonable request.
